# Suboptimal B-cell depletion is associated with progression independent of relapse activity in multiple sclerosis patients treated with ocrelizumab

**DOI:** 10.1177/13524585251329849

**Published:** 2025-04-03

**Authors:** Lisa Revie, Annika Jürjens, Maria Eveslage, Susan Trümpelmann, Valerie Teschner, Andreas Schulte-Mecklenbeck, Catharina C Gross, Jan D Lünemann, Jan Grosch, Catharina Korsukewitz, Heinz Wiendl, Luisa Klotz

**Affiliations:** Department of Neurology With Institute of Translational Neurology, University Hospital Münster, Münster, Germany; Department of Neurology With Institute of Translational Neurology, University Hospital Münster, Münster, Germany; Institute of Biostatistics and Clinical Research, University of Münster, Münster, Germany; Department of Neurology With Institute of Translational Neurology, University Hospital Münster, Münster, Germany; Department of Neurology With Institute of Translational Neurology, University Hospital Münster, Münster, Germany; Department of Neurology With Institute of Translational Neurology, University Hospital Münster, Münster, Germany; Department of Neurology With Institute of Translational Neurology, University Hospital Münster, Münster, Germany; Department of Neurology With Institute of Translational Neurology, University Hospital Münster, Münster, Germany; Department of Neurology With Institute of Translational Neurology, University Hospital Münster, Münster, Germany; Department of Neurology With Institute of Translational Neurology, University Hospital Münster, Münster, Germany; Department of Neurology With Institute of Translational Neurology, University Hospital Münster, Münster, Germany; Department of Neurology With Institute of Translational Neurology, University Hospital Münster, Münster, Germany

**Keywords:** Multiple sclerosis, PIRA, progression, ocrelizumab, B-cell depletion

## Abstract

**Background:**

While treatment with ocrelizumab has proven effective in preventing relapse-associated worsening (RAW) in relapsing multiple sclerosis (RMS), a significant number of patients experience progression independent of relapse activity (PIRA).

**Objectives:**

To investigate the association between B-cell depletion status and the risk of disability accumulation in RMS patients receiving ocrelizumab treatment.

**Methods:**

In this monocentric cohort study of 148 RMS patients (2017–2023), we categorized participants into three groups: no evidence of disease activity (NEDA), evidence of disease activity (EDA), and PIRA. B-cell counts were measured every 6–12 months, with suboptimal depletion defined as ⩾10 CD19+ B-cells/µL. Logistic regression and Cox proportional hazards models analyzed the relationship between B-cell depletion and disability progression.

**Results:**

Of 148 patients, 70 (47%) achieved NEDA, 51 (34%) showed EDA, and 25 (17%) developed PIRA. NEDA patients demonstrated significantly lower B-cell counts compared to EDA (*p* < 0.01) and PIRA (*p* < 0.001) groups. Insufficient B-cell depletion was the strongest PIRA predictor (OR 3.73, 95% CI: 2.50–5.43, *p* < 0.001) and increased EDSS progression risk (HR 0.50, 95% CI: 0.26–0.97, *p* = 0.039).

**Conclusions:**

PIRA occurs in the context of suboptimal B-cell depletion in RMS patients, highlighting the need for close monitoring and potential adjustment of infusion intervals.

## Introduction

In relapsing multiple sclerosis (RMS), disability accumulation can manifest through relapse-associated worsening (RAW) or progression that occurs independent of relapse activity (PIRA). While the prevailing consensus was previously anchored in the concept that RAW constituted the primary cause of disease progression at least in the relapsing-remitting phase of multiple sclerosis, recent studies have posed a significant challenge to this hypothesis. Emerging evidence from several independent studies now suggests that PIRA is the main driver of disability progression even in early stages of RMS.^1–4^

While various studies have proposed definitions for PIRA based on clinical trial data, there is still no universally accepted definition for use in real-world clinical settings.^5^ The variability in patient characteristics and treatment responses outside of controlled environments underscores the need for a standardized approach that can be applied consistently across diverse clinical populations, ensuring more accurate tracking of disability progression in everyday practice.

B-cell depleting therapies such as ocrelizumab have demonstrated high efficacy in reducing relapse rates, yet PIRA occurs in a notable proportion of treated patients.^1,6^ Lately, it has been proposed that the dosing of ocrelizumab may be adequate for managing relapse activity but insufficient for controlling disease progression.^7^

This study aimed to investigate whether there was an association between the risk of disability accumulation and extent of B-cell depletion in RMS patients undergoing ocrelizumab treatment.

## Methods

### Patient data collection

In this monocentric cohort study, we retrospectively collected longitudinal clinical and MRI data by reviewing patient charts of the Department of Neurology, University Hospital Münster, Germany, covering the period from 2017 to 2023. The data were assessed during clinical routine visits and retrospectively collected from the hospital information system. Inclusion criteria were a diagnosis of RMS according to the 2017 revised McDonald criteria^8^ with an active disease course receiving treatment with ocrelizumab in a 6-month protocol independent from B-cell counts, and longitudinal data on Expanded Disability Status Scale (EDSS) as well as relapse activity for ⩾12 months. Ethics approval was obtained from the ethics committee of the University of Münster, and all patients provided written informed consent.

We retrospectively collected baseline demographics including weight and height as well as longitudinal data on EDSS and relapse activity documented by the clinician every 6 months at the routine infusion visits. In addition, Brain MRI scans were performed as part of standard care at minimum 12-month intervals. MRI activity was defined as the presence of gadolinium-enhancing lesions on T1-weighted images or the appearance of new or enlarging T2 lesions compared to prior MRI scans. Due to the variability and lack of standardization in MRI protocols performed on different scanners in routine clinical practice, our analysis was restricted to detecting inflammatory lesions.

In the real-world setting of routine visits for ocrelizumab infusions, no objective clinical assessments of disease progression other than the EDSS were performed. Nevertheless, we screened patient records for patient-reported symptoms of progression that did not necessarily lead to EDSS deterioration (i.e. reduction of walking distance, cognitive fatigue, motor fatigue, spasticity or pain, cognitive deterioration, or micturition disturbances), and documented these at all time points at 6-month intervals.

Data on absolute and relative CD19+ B-cell counts were obtained every 6–12 months from routinely performed flow cytometry immunophenotyping using EDTA-anticoagulated blood, conducted at the end of the 6-month treatment-free interval between ocrelizumab infusions. For analyses performed at our clinic, samples were stained according to the manufacturer’s protocol using the BD Multitest 6 Color TBNK antibody mixture (CD3 FITC, CD16 PE, CD56 PE, CD45 PerCP-Cy5.5, CD4 PE-Cy7, CD19 APC, CD8 APC-Cy7, BD Biosciences). Flow cytometry was performed on the BD FACSCanto II (BD Biosciences), sample analysis was performed with FACS Diva 2.0 software and BD FACSLyric, with FACSSuite software (BD Biosciences). Automatic gating with manual adjustment was performed for CD19+ B-cells (CD3^-^CD16^-^CD56^-^CD19 ^+^; reference values: absolute B-cells 100–800 cells/µL, relative B-cells 0.07–0.23). However, since the study was conducted in a real-world setting, not all routine assessments of lymphocyte differentiation were conducted at our clinic but were partially performed at external laboratories. The reference values remained the same.

The threshold for suboptimal B-cell depletion was set at ⩾10 CD19+ B-cells/µL. This cut-off value has been validated in clinical studies, where B-cell counts below this threshold indicate effective depletion, while counts above 10 B-cells/µL have been used as a criterion for re-dosing decisions.^9,10^

Confirmed disability accumulation (CDA) was defined as an increase in the EDSS score of ⩾1.5 points from an EDSS of 0, ⩾1.0 point from an EDSS of 1.0 to 5.0, or ⩾0.5 point from an EDSS score of ⩾5.5 sustained for ⩾6 months. PIRA was defined as CDA that occurred independently or at least >90 days after and >30 days before onset of a relapse. RAW was defined as CDA <90 days after or <30 days before onset of a relapse. A status of no evidence of disease activity (NEDA) was defined as no CDA, relapse, or MRI activity as well as no patient-reported clinical signs of disease progression.

Previous studies have demonstrated that the body mass index (BMI) significantly influences treatment outcomes in patients receiving ocrelizumab.^11^ A key analysis of ocrelizumab efficacy revealed significant treatment-by-subgroup interactions for both 12 and 24 weeks confirmed disability progression between patients with different BMI levels. Given these established associations between BMI and treatment response, we included BMI as an important covariate in our analysis to account for its potential impact on B-cell depletion patterns and clinical outcomes.^12^

### Statistical analysis

Statistical analyses were performed using R software (version 4.2.2). Due to non-normal distribution of B-cell counts across groups, as confirmed by Shapiro–Wilk tests, non-parametric methods were employed. Initial group comparisons between NEDA, EDA, and PIRA patients were conducted using Kruskal–Wallis tests, followed by post hoc pairwise Wilcoxon tests with Bonferroni correction for multiple comparisons. To account for potential confounding variables and repeated measurements, we additionally performed linear mixed-effects models, adjusting for age, BMI, sex, EDSS and treatment duration. The model included random effects to account for patient-specific variations, and variance inflation factors were calculated to check for multicollinearity.

We employed a comprehensive logistic regression model to examine factors associated with PIRA versus NEDA status. The binary outcome variable was modeled using logistic regression, incorporating key predictors including age, sex, BMI, EDSS, treatment duration, and B-cell depletion status. The model’s statistical measures included odds ratios (OR) with corresponding 95% confidence intervals and *p*-values based on *z*-test statistics. For continuous variables, odds ratios represent the change in odds for a one-unit increase, while for categorical variables, they indicate odds relative to the reference category. Results were visualized using forest plots displaying point estimates, confidence intervals, and significance levels for each predictor.

For our survival analysis, we employed a Cox proportional hazards model to examine the relationship between B-cell depletion and disability progression, while accounting for both time-dependent and fixed covariates. The analysis focused on time to EDSS progression as the primary outcome variable. Our model incorporated B-cell depletion status as a time-dependent covariate, while controlling for baseline characteristics including age, sex, BMI, and baseline EDSS and treatment duration as fixed covariates.^13^ Since the Kaplan–Meier plot is not suitable for displaying time-dependent covariates, we performed a supplementary landmark analysis, where the B-cell status of <10/µL or ⩾10/µL was determined from the 6-month landmark after the start of therapy. The landmark method allows examining survival based on covariate values at pre-specified time points, avoiding the limitations of Kaplan–Meier with time-dependent variables.^14^ Statistical significance was set at *p* < 0.05. Due to the small sample size (*n* = 2), RAW patients were excluded from the analysis.

## Results

We included a total of 148 patients diagnosed with RMS, with a median ocrelizumab treatment duration of 24.4 months corresponding to at least five infusion cycles. Notably, 70 (47%) patients achieved NEDA status, reflecting a lack of relapses, disability progression, or new MRI activity during treatment. 25 (17%) patients exhibited PIRA, while only two (1%) patients experienced RAW. Interestingly, 51 (34%) patients from the overall cohort reported clinical signs of progression during routine clinical visits without meeting the criteria for CDA. However, these patients did not fit the criteria of NEDA since it required lack of patient-reported progression symptoms and any EDSS deterioration. Therefore, we classified this group as patients with evidence of disease activity (EDA) without fulfilling PIRA criteria. 110 (74%) patients did receive a disease modifying therapy previous to ocrelizumab, however none of these patients were treated with a B-cell depleting therapy beforehand. The between group analysis revealed a significant difference for age at baseline and treatment duration between NEDA versus EDA and PIRA, highlighting the need for adjustment for these covariates in further analyses. Table 1 depicts the demographic and clinical characteristics of the included patients and subgroups.

**Table 1.** Patient characteristics.

	ALL median or *n* (IQR or %)	NEDA median or *n* (IQR or %)	EDA median or *n* (IQR or %)	PIRA median or *n* (IQR or %)	*p*-value
**no. of patients**	148	70	51	25	-
**age at baseline (years)**	39.3 (18.3)	33.1 (19.0)	43.0 (13.9)	46.1 (14.8)	0.002^†^*
**female sex**	101 (68.2%)	52 (74.2%)	30 (58.8%)	18 (72.0%)	0.276^‡^
**treatment-naïve at baseline**	38 (25.7%)	18 (25.7%)	12 (23.5%)	8 (32.0%)	0.805^‡^
**treatment duration (months)**	24.4 (25.9)	23.5 (22.6)	36.1 (25.0)	25.1 (31.7)	0.016^†^**
**EDSS at baseline**	2.5 (2.5)	2.5 (2.0)	2.5 (2.0)	3.0 (3.5)	0.137^†^
**BMI at baseline (kg/m^2^)**	24.6 (4.9)	24.2 (5.4)	24.9 (4.1)	26.2 (7.5)	0.073^†^

Due to the small number, the RAW subgroup of two patients was not included as a separate column. Abbreviations: NEDA = no evidence of disease activity; EDA = evidence of disease activity; PIRA = progression independent of relapse activity; IQR = interquartile range. Values for continuous variables are presented as median (IQR). Categorical variables are presented as *n* (%). Statistical tests: ^†^Kruskal–Wallis test followed post hoc comparisons performed using Dunn’s test with Bonferroni correction; ^‡^Chi-square test. Significant *p*-value < 0.05.

* NEDA versus EDA: *p* = 0.008; NEDA versus PIRA: *p* = 0.015.

** NEDA versus EDA: *p* = 0.003; NEDA versus PIRA: *p* = 0.034.

**Figure 1.**
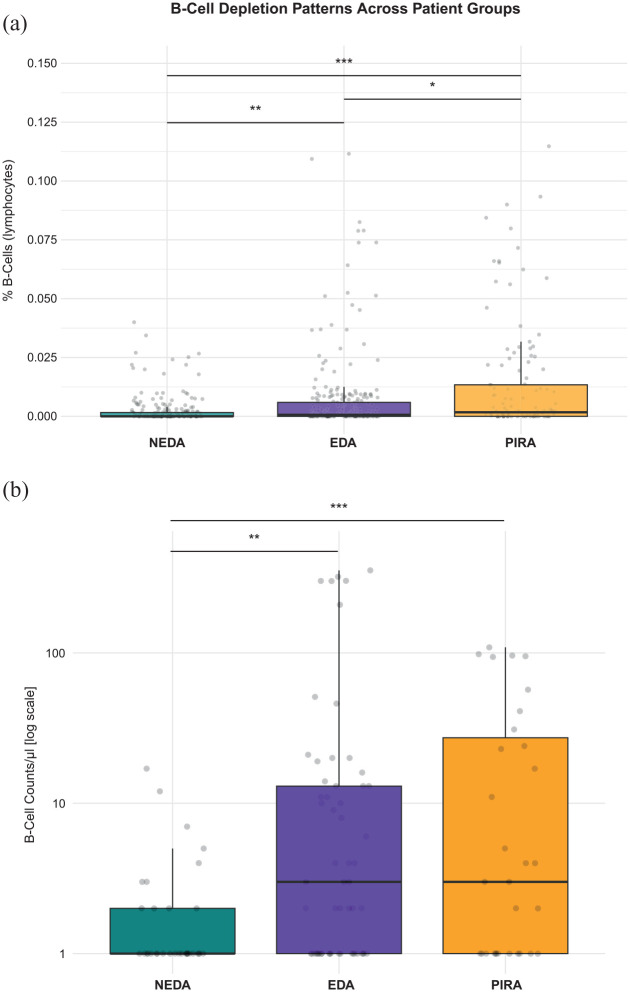
**(a)** Percentage of B-cells within lymphocytes are compared between three patient groups: NEDA, *n* = 70; median = 0.0%, IQR = 0.17%, EDA, *n* = 51; median = 0.07%, IQR = 0.63%, and PIRA, *n* = 25; median = 0.17%, IQR = 1.34%. The boxplot displays the median (horizontal line), interquartile range (box), and individual data points (jittered dots). Statistical significance was determined using pairwise Wilcoxon rank-sum tests with Bonferroni correction for multiple comparisons with adjustment for covariates age, sex, BMI, baseline EDSS and treatment duration in a linear mixed effect model. NEDA patients showed significantly lower B-cell counts compared to both EDA (*p* < 0.01) and PIRA (*p* < 0.001) groups, while EDA patients maintained intermediate levels, significantly different from PIRA patients (*p* < 0.05). These findings suggest a gradient of B-cell depletion effectiveness across the three patient groups, with NEDA patients showing the most effective B-cell depletion. **(b)** Absolute B-cell counts. Median (IQR) values: NEDA: 0 (0–1) cells/µL; EDA: 2 (0–12) cells/µL; and PIRA: 2 (0–26.5) cells/µL. Statistical significance was assessed using the Kruskal–Wallis test (*p* < 0.001) followed by pairwise Wilcoxon rank-sum tests with Bonferroni correction for multiple comparisons with adjustment for covariates age, sex, BMI, baseline EDSS and treatment duration in a linear mixed effect model. Significant differences were found between NEDA versus EDA (*p* < 0.01) and NEDA versus PIRA (*p* < 0.001). Y-axis is shown on a log10 scale. Significance levels are indicated by asterisks: *p < 0.05, **p < 0.01, ***p < 0.001.

To evaluate whether there is an association between the disease status and B-cell counts, we initiated our analysis by comparing percentage of B-cells within lymphocytes as well as absolute B-cell counts/µL between our defined groups NEDA, EDA and PIRA adjusted for age, sex, BMI, baseline EDSS and treatment duration. Remarkably, the overall and in between group analysis showed significant differences between all three groups. B-cell depletion was most pronounced in NEDA patients, who demonstrated significantly reduced percentages of B-cells and absolute B-cells/µL compared to both EDA (*p* < 0.01) and PIRA (*p* < 0.001) groups. A gradient pattern emerged, where EDA patients exhibited intermediate B-cell levels, while PIRA patients showed the highest B-cell counts. These differences were statistically significant between all groups for percentage of B-cells within lymphocytes (EDA vs PIRA: *p* < 0.05), suggesting that the degree of B-cell depletion may be associated with disease progression (Figure 1).

**Figure 2.**
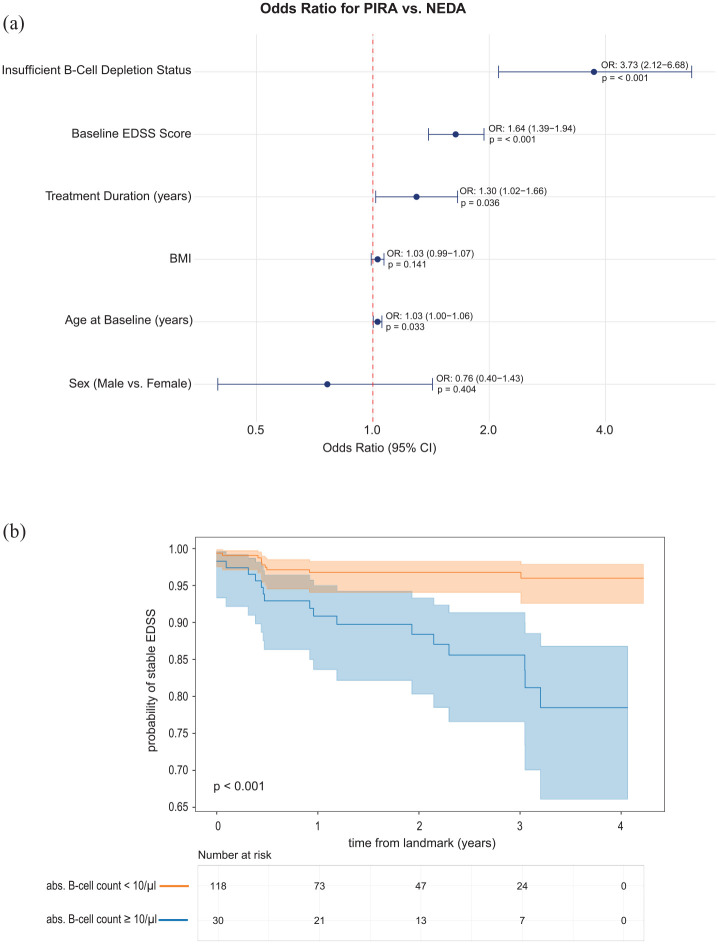
**(a)** Multivariable logistic regression analysis comparing PIRA versus NEDA status, with adjustment for age and treatment duration. The forest plot displays adjusted odds ratios (OR) with 95% confidence intervals (CI). Values greater than 1.0 indicate increased odds of PIRA, while values less than 1.0 indicate decreased odds of PIRA. Significant associations persisted for age (OR: 1.03; 95% CI: 1.00–1.06), EDSS score (OR: 1.64; 95% CI: 1.39–1.94), treatment duration (OR: 1.30; 95% CI: 1.02–1.66), and insufficient B-cell depletion status < 10 B-cells/µL (OR: 3.73; 95% CI: 2.50–5.43). Sex and BMI showed no significant association. The red dashed line represents an OR of 1.0 (no association). *p*-values < 0.05 were considered statistically significant. EDSS: Expanded Disability Status Scale; BMI: body mass index. (**b)** Kaplan–Meier curve depicting probability of stable EDSS over time in years from 6-month landmark from therapy start (y-axis) in RMS patients, segmented by B-cell count <10/µL (orange) and ⩾10/µL (blue) adjusted for covariates age, BMI, sex, baseline EDSS and treatment duration. The y-axis shows the survival probability, steps representing an EDSS deterioration of ⩾0.5. points. Cox Proportional Hazards model revealed that B-cell depletion < 10/µL was associated with a higher probability of stable EDSS over time from landmark compared to suboptimal B-cell depletion (HR = 0.22, SE = 0.38, 95% CI [0.10, 0.47], *p*-value < 0.001).

The logistic regression analysis comparing PIRA versus NEDA status adjusted for covariates revealed several significant predictors of disease progression. Higher age was associated with increased odds of PIRA (OR 1.03, 95% CI: 1.00–1.06, *p* = 0.033), with each additional year increasing the probability by 3%. Baseline EDSS emerged as a strong predictor, with each point increase associated with 64% higher odds of PIRA (OR 1.64, 95% CI: 1.39–1.94, *p* < 0.001). Even after adjustment, treatment duration also showed a significant effect, with longer duration associated with 30% increased odds of PIRA per year (OR 1.30, 95% CI: 1.02–1.66, *p* = 0.036). Most notably, insufficient B-cell depletion ⩾10/µL was the strongest predictor, associated with nearly four-fold higher odds of PIRA (OR 3.73, 95% CI: 2.50–5.43, *p* < 0.001). Neither sex nor BMI showed significant associations with PIRA status in this analysis (Figure 2(a)).

The results of our survival analysis revealed a significant protective effect of B-cell depletion, with a hazard ratio of 0.50 (95% CI: 0.26–0.97, *p* = 0.039), indicating that patients with adequate B-cell depletion had a 50% lower risk of EDSS progression compared to those without sufficient depletion. This finding remained significant after adjusting for other covariates in the model. Non-time-dependent covariates, including sex, BMI, and baseline EDSS, did not show significant associations with progression risk in the adjusted analysis. In addition, Cox regression analysis showed that B-cell depletion ⩽ 10/µL was associated with a higher probability of stable EDSS over time from the landmark of 6 months compared to suboptimal B-cell depletion (HR = 0.22, 95% CI: 0.10, 0.47, *p*-value < 0.001) (Figure 2(b)).

## Discussion

Our findings carry two important implications: First, they validate, within a real-world treatment setting of B-cell depletion, the hitherto underestimated role of relapse-independent disease progression in the context of sufficient control of focal inflammatory activity. Second, our results unveil a discernible relationship between the degree of B-cell depletion and disease progression in individuals with RMS.

The relevance of PIRA as a primary driver of disability accumulation has gained recognition in recent studies investigating various cohorts of early RMS providing evidence of early occurrence of PIRA within the MS disease continuum.^3,15^ This acknowledgment extends to clinical trials utilizing B-cell-depleting therapies.^1^ The identification of PIRA as an early key contributor to disability accumulation has prompted a reassessment of our conceptual framework for RMS.^2^ Furthermore, it challenges the prevailing notion that successful control of focal inflammatory activity is the primary key treatment goal in multiple sclerosis, as PIRA is found to occur even in a sizable proportion of patients undergoing high-efficacy therapies.^4^ A post hoc analysis of data from the OPERA trials indicates that while ocrelizumab dosing might be effective in controlling focal inflammatory activity, it may not be sufficient for controlling relapse-independent disease progression.^1^ Two potential explanations emerge: either PIRA is driven by pathomechanisms unrelated to B-cells, or the standard dosing of ocrelizumab may not adequately deplete central nervous system (CNS) localized B-cells to control disease progression. Supporting the latter scenario, our analysis established a significant correlation between incomplete B-cell depletion and occurrence of PIRA in our real-world cohort. Notably, insufficient B-cell depletion emerged as the strongest predictor of PIRA, associated with nearly four-fold higher odds compared to adequate depletion. Our analysis identified several significant predictors of disease progression, including age, baseline EDSS, and treatment duration. The finding that each additional year of age increased PIRA probability by 3% aligns with previous studies showing age-related effects on disease progression.^3^ The strong association between baseline EDSS and progression risk (64% higher odds per point increase) emphasizes the importance of early intervention. Our survival analyses underscore the potential impact of B-cell depletion on the long-term disability outcomes of RMS patients. The increased probability of experiencing an EDSS worsening over time in non-B-cell depleted patients emphasizes the importance of B-cell dynamics in shaping the trajectory of disability progression.

Furthermore, the identification of a distinct EDA group, showing intermediate B-cell levels between NEDA and PIRA patients, suggests that current PIRA definitions may not fully capture the spectrum of disease progression in clinical practice. While these patients did not meet formal PIRA criteria, they demonstrated evidence of disease activity through patient-reported symptoms and showed different B-cell depletion patterns compared to both NEDA and PIRA groups. This observation underscores the need for more nuanced and practical definitions of disease progression that can be readily applied in routine clinical care. Moreover, it should be discussed whether the definition of PIRA is purely based on clinical observation or whether MRI activity also plays a supportive role, as a recent meta-analysis showed that in at least 45% of cases, PIRA was associated with concurrent focal MRI activity.^16^

Acknowledging the need for replication of our findings in an independent cohort, we recognize the necessity to reconsider the concept of B-cell depletion in multiple sclerosis. This may involve exploring higher doses of ocrelizumab, currently under investigation in two clinical trials in RMS (NCT04544436) and primary progressive MS (NCT04548999) and/or optimizing individual B-cell depletion by adjusting the dosing intervals according to the extent of depletion.

Several aspects warrant acknowledgment: First, the retrospective, single-center design may limit generalizability, and the varying age and treatment durations between groups required statistical adjustment. Second, our data as well as data from previous research indicate that determination of progression solely based on the EDSS might not capture clinically relevant progression in many cases due to a low interrater reliability in lower EDSS scores and due to underscoring of non-walking associated symptoms at higher EDSS scores. Third, the lack of frequent longitudinal data on B-cell counts during depletion hinders the distinction between effects due to insufficient depletion or to premature repopulation, necessitating further investigation in subsequent studies. We also acknowledge that our observed PIRA rate of 17% appears higher than the 3.1% annual rate reported in a recent meta-analysis.^5^ This difference likely reflects the real-world nature of our study, including a more heterogeneous patient population with relatively high baseline disability (median EDSS 2.5). Higher baseline EDSS was significantly associated with increased PIRA risk (OR 1.64, *p* < 0.001), potentially contributing to the higher progression rate observed in our cohort compared to controlled clinical trials. Finally, we must note that not all analyses of CD19+ B-cell counts could be performed using the standardized flow cytometry procedure at our clinic and were partially conducted by external laboratories. As a result, we acknowledge that variability and reduced comparability of results may arise due to the use of different equipment.

In summary, our study provides evidence that PIRA occurs in the context of suboptimal B-cell depletion in a relevant proportion of patients with RMS. This underscores the importance of closely monitoring B-cell counts and potentially adjusting infusion intervals. Moving forward, a nuanced understanding of B-cell dynamics will be pivotal for refining treatment strategies and improving outcomes for patients with RMS.
